# Global nutritional challenges of reformulated food: A review

**DOI:** 10.1002/fsn3.3286

**Published:** 2023-03-06

**Authors:** Helen Onyeaka, Ogueri Nwaiwu, KeChrist Obileke, Taghi Miri, Zainab T. Al‐Sharify

**Affiliations:** ^1^ School of Chemical Engineering University of Birmingham, Edgbaston Birmingham UK; ^2^ Faculty of Science and Agriculture University of Fort Hare Alice South Africa; ^3^ Department of Environmental Engineering, College of Engineering University of Al‐Mustansiriya Baghdad Iraq

**Keywords:** fat, food reformulation, fortification, functional food, nutrition, process formulation, salt, sugar

## Abstract

Food reformulation, the process of redesigning processed food products to make them healthier, is considered a crucial step in the fight against noncommunicable diseases. The reasons for reformulating food vary, with a common focus on reducing the levels of harmful substances, such as fats, sugars, and salts. Although this topic is broad, this review aims to shed light on the current challenges faced in the reformulation of food and to explore different approaches that can be taken to overcome these challenges. The review highlights the perception of consumer risk, the reasons for reformulating food, and the challenges involved. The review also emphasizes the importance of fortifying artisanal food processing and modifying microbial fermentation in order to meet the nutrient requirements of people in developing countries. The literature suggests that while the traditional reductionist approach remains relevant and yields quicker results, the food matrix approach, which involves engineering food microstructure, is a more complex process that may take longer to implement in developing economies. The findings of the review indicate that food reformulation policies are more likely to succeed if the private sector collaborates with or responds to the government regulatory process, and further research is conducted to establish newly developed reformulation concepts from different countries. In conclusion, food reformulation holds great promise in reducing the burden of noncommunicable diseases and improving the health of people around the world.

## Graphical abstract

Redeveloping an existing processed food product to make it healthier is the focus of food reformulation. However, the approaches for food reformulation are diverse, and there is a consensus that the main concerns are fats, sugars, and salts. Food reformulation policy would certainly succeed if the private sector works in tandem with and/or responds to government pressure.

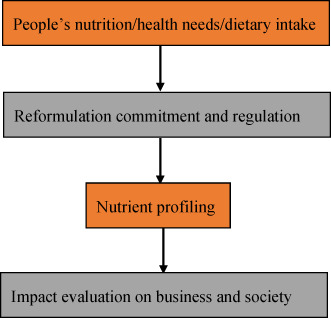

## 1 INTRODUCTION

According to a WHO report (WHO, 2020), 38.2 million children under the age of 5 were estimated to be overweight or obese in 2019. Globally, 39% of adults are overweight and 13% are obese, and in most countries of the world, being overweight and obese are more significant problems than being underweight. Overweight and obesity, once thought to be a problem only in high‐income countries, are now on the rise in low‐ and middle‐income countries. In 2016, over 340 million children and adolescents aged 5–19 were overweight or obese. Obesity and overweight among children and adolescents aged 5–19 years old have increased dramatically from 4% in 1975 to just over 18% in 2016. In 2016, more than 1.9 billion adults were overweight and 600 million of them were obese. Four interdependent factors, namely the food environment, psychological influences, physical activity, and an individual's biology, influence obesity (Harastani et al., 2020; Vandenbroeck et al., 2007).

Obesity and overweight are caused by an energy imbalance between calories consumed and calories expended. Globally, the causes of obesity have been attributed to the increased intake of energy‐dense foods that are high in fat and sugars, an increase in physical inactivity due to the increasingly sedentary nature of many forms of work, changing modes of transportation, and increasing urbanization (WHO, 2020). These factors can be controlled, and as such, obesity can be prevented (Hall, 2018). Pineda et al. (2018) reported that obesity is expected to rise globally if no control measures are taken. We live in a world where an unsustainable paradox exists: for every person malnourished, there are two who are overweight or obese. Obesity is a major public health concern because it is linked to several chronic diseases, including diabetes, cardiovascular disease, breast cancer, and colorectal cancer. High intakes of energy, salt, saturated fat, and sugar lead to a higher risk of chronic diseases like heart disease, cancer, and diabetes (WHO, 2003). The significant rise in obesity rates and dependence on convenience‐processed foods and the attendant noncommunicable diseases that follow has led to the push for food reformulation in the last two decades. The redesigning of an existing processed food product to make it healthier is known as food reformulation.

A recent study conducted by Lee et al. (2022) focused on the association between saturated fatty acids and noncommunicable diseases. In their study, it was reported that saturated fats from animal sources decreased while polyunsaturated fats from vegetable oils rose. In this case, noncommunicable diseases (NCD) became prevalent in the 20th century with increased consumption of processed food (sugar, refined flour, rice, and vegetable oils). The increased media attention and public demand for reformulation are expected to improve nutrition and public health. Hence, reformulation can be done to meet changing consumer preferences and tastes for healthier products, decrease cost and maximize profit, comply with formal regulatory directives, and tap into new consumer markets to offset declining sales (Fanzo & McLaren, 2020). According to Federici et al. (2019), the reformulation of processed food has the potential and capacity to improve the population's diet. In their study, a model was developed that revealed that food reform is relevant in improving diets and population health. Many governments have adopted reformulation policies to protect the population from noncommunicable diseases (NCDs), focusing primarily on reducing sodium or trans fatty acids (TFA) in foods. A report highlighted that the focus of reformulation policies has shifted to reducing added sugar and energy (Tedstone et al., 2018). For example, the English government released “Childhood Obesity: A Plan for Action: in August 2016 and Public Health England (PHE) was given the task of overseeing a sugar reduction program. This tasked the food industry to reduce 20% sugar levels by 2020 in categories contributing to the intake of children under 18. In the first year of the program, the industry was also challenged to achieve a 5% reduction (Tedstone et al., 2018). For the food categories included in the program, PHE published in March 2017 guidelines for total sugar levels per 100 g and calorie content of products likely to be consumed on a single occasion. These were created to assist the industry in meeting its 20% reduction goal. Another example is the Danish Legislation on Trans Fats in 2004, which implemented a maximum of 2% of total oil or fat sold directly to consumers or used as ingredients in all processed foods (Traill et al., 2012). Industries are willing to work with the government and advocates to make changes.

Companies are responding as more products with less sugar are offered by soda manufacturers. In 2018, Nestlé announced that some of its bars would reduce their sugar level by 30%. Even candy companies are reformulating their treats (Friedman, 2019). Also, a salt reduction target has been launched to drastically reduce salts in the United Kingdom by 2024 (PHE, 2020). To solve the problems highlighted above, food reformulation has been on the agenda of communities and food companies to increase their food supplies and reduce energy density, trans fat, saturated fat, sodium, and added sugar in food products. In another development, the world's action on salt, sugar, and health commits to improving the health of the population globally by achieving a reduction in salt and sugar intake (WASSH, 2022). Attention has been paid to measures of promoting processed food reformulation toward healthier alternatives (Griffith et al., 2017). However, the reformulation will be successful only when replenished foods are not only healthy in the diet but also of high quality and good texture, safe, delicious, and affordable (Jiménez‐Colmenero et al., 2010). Several studies have been conducted on food reformation that covers both technical aspects of sucrose or saturated fat reductions (Jiménez‐Colmenero et al., 2010) and consumer aspects, such as food choices and human behavior (Grunert et al., 2012).

Food reformulation requires taking into consideration several factors which may include the effect of the water–energy–food nexus (Peña‐Torres et al., 2022), lowering of the digestibility of starch caused by the physical barriers of dietary fiber (Zhang, Noort, et al., 2022; Zhang, Sun, et al., 2022), future fortification with the right ratio of omega acids (Patel et al., 2022), and taking into consideration the different models of lowering salt content (Vinitha et al., 2022). Other factors that can support reformulation include food labeling (Lisa Clodoveo et al., 2022) and engineering microstructure (Aguilera, 2022), which may include taking care of inverse problems (Reddy et al., 2022) that often occurs in food engineering. The use of nanotechnology can also aid reformulation even though there are safety concerns (Onyeaka et al., 2022). Knowledge of the interactions of proteins and flavor (Reineccius, 2022) and the design of functional foods using agroindustrial valorized wastes from food processing (Gedi et al., 2020) can also contribute toward the design of foods that can meet customers' reformulation needs. To highlight global nutritional reformulation challenges, the purpose of this narrative review was aimed to obtain an overview of the aspects involved in reformulating foods, perception of consumer risk, challenges and product processability, and its limits to exploring food reformulation. The impact of the key food groups, namely salts, sugar, and fats, was reviewed thoroughly. The acceptable intake rate on a global scale was listed for different types of reformulated food products, which include bread, cheese, meat, and soups miscellaneous products. A broad strategy for salt, sugar, and fat reduction was recommended to avoid obesity and other health diseases. In addition, the role of consumers, food companies, government policies, and other agents were pointed out and it was suggested that a different approach should be used for people living in rural areas in developing countries by focusing on the reformulation of artisanal food by fortification.

## 2 REVIEW METHODS

The method employed in this review involved conducting a narrative and bibliography review of various databases, including PubMed, Science Direct, Web of Science, Scopus, and Google Scholar. The search was conducted using relevant keywords related to food reformulation. Duplicate reports were eliminated, and the common knowledge about reformulating food and the main food groups associated with noncommunicable diseases (namely salts, sugars, and fats) was documented.

In addition, relevant data and statistics from World Health Organization (WHO) reports were collected and analyzed, and the current trends and global challenges of food reformulation were highlighted. This information helped to provide a comprehensive understanding of the subject and its impact on the global community. The main highlights include:

- Global nutritional challenges posed by reformulated food and the efforts to address them in a broad manner. The article's focus is on reducing salt, sugar, and fat in food products, as these are the three main health concerns globally.
- Efforts to reduce salt intake and its benefits, including reduced blood pressure and cardiovascular diseases, were also highlighted.
- The importance of considering several factors in food reformulation, such as the water–energy–food nexus, fortification, especially with the right ratio of omega acids, food labeling, and microstructure engineering of the food matrix.

## 3 SALTS, SUGARS, AND FATS

The three main health concerns across the globe are salts, sugars, and fats, and hence there is a drive to lower their content in food.

### 3.1 Salts

The system in the body relies on the balance of electrolytes such as sodium, potassium, and chlorine. However, sodium is a dietary essential but is far lower than the current intake. There has been concern about the rate of salt intake and blood pressure which was triggered during the Food Standard Agency Salt Campaign in England (Wyness et al., 2012). On a global scale (He et al., 2019), there are indications that blood pressure and cardiovascular diseases have been reduced due to a reduction in salt intake (Wilson et al., 2012, Table 1). In addition, renal diseases can also be reduced with a reduction in salt consumption (Cook et al., 2007; Elliott & Brown, 2007; Habib et al., 2022; He & MacGregor, 2010; Jones‐Burton et al., 2006). Jaenke et al. (2017) studied salt reduction in reformulated food. They reviewed 50 investigations on bread, cheese, meat, soups, and miscellaneous products such as potato and tortilla chips (Adams et al., 1995), biscuit (Vázquez et al. 2009), and sweet pickles (Wyatt, 1983). The salt reduction was categorized into four groups: less than 40% and no more than 80%. They concluded that salt could be reduced in the formulated meat by 70% and in bread by 40%, which will perform better in a healthier food supply.

**TABLE 1:** Effects of salt in selected foods (Briggs et al., 2017).

Foods	Effects of salt	Studies
Bread and wheat	Control the growth of yeast and fermentation; make gluten more stable; and assist in preservation and reduction of spoilage	Adams et al. (1995), Bolhuis et al. (2011), Ferrante et al. (2011), Girgis et al. (2003), Hellemann et al. (1990) La Croix et al. (2014), Miller and Jeong (2014), Noort et al. (2012) White and Wyatt (1983)
Cheese	Regulate the activity of the starter culture; modify enzymes activities; and has a direct effect on water content.	Czarnacka‐Szymani and Jezewska‐Zychowicz (2015), Drake et al. (2011) Ganesan et al. (2014), Karahadian and Lindsay (1984) Lindsay et al. (1982) Schroeder et al. (1988) and Wyatt (1983))
Meat	Contributes sensory properties and textural properties through the process of solubilization of myofibrillar proteins and also safety	Corral et al. (2013), Galvão et al. (2014), Lopez et al. (2012), Pietrasik and Gaudette (2014), Saha et al. (2009), Sofos (1983) Tobin et al. (2012) Tobin et al. (2013) Chicken broth, Ghawi et al. (2014)
Vegetables and Snacks	Create hard bite texture and expansion during cooking	Kanavouras et al. (2005), Vázquez et al. (2009), Wyatt (1983), Methven et al. (2012) Mitchell et al. (2013), Willems et al. (2014)

Studies have shown that there is a relationship between the quantity of salt intake and blood pressure (He et al., 2013) in both animal models and human studies (He & MacGregor, 2010). It is recommended globally that prevention and control of high blood pressure can be taken care of through moderate reduction in salt intake. Despite the benefits of sodium in regulating, osmotic equilibrium and pH, its excess leads to fluid retention and subsequent blood pressure (Appel et al., 2011). For stroke, it is regarded as one of the silent leading causes of mortality. The reduction in salt intake to approximately 1500 mg/day reduces the risk of stroke as well as the overall morbidity and mortality of cardiovascular diseases and stroke (Ekaterina & Feng, 2013).

Salt is used as a preservative in foods to control the water activities and growth of food poisoning and spoilage organism, and it is also effective in flavor perception. Despite the effects or properties of salt in foods (influencing taste, texture, shelf life, and food safety), there have been advances in reducing salt (Obileke et al., 2022; Wilson et al., 2012). However, each food has its challenges, as presented in Table 2. For example, the Federation of Bakers in England recorded a reduction of 23% in the salt content of bread since 2004. It was estimated that a total reduction of 40% had taken place from the 1980s to the 1990s. From Table 2, it can be concluded that the major challenge for salt reduction in foods is to ensure that reductions do not outpace consumer expectations from a flavor point of view, as this would be counterproductive. By counterproductive, it deals with the fall in the sale which may affect the company's profits. The strategic approaches and current position for salt reduction as part of food reformulation have been outlined (Table 3). The daily salt intake from different countries varies as reported by the national dietary intake surveys suggesting that across developed countries, the highest's intakes are from cereals, cereal products, and bread (Bates et al., 2014; Bureau of Statistics, 2014; Doyle & Glass, 2010; Elliott & Brown, 2007; Health Canada, 2010). Other sources include meat, milk, and snacks (Webster et al., 2014). In Asian countries, salt intake is high in cooked food (Anderson et al., 2010; WHO, 2011). However, recommendations from WHO in 2012 aimed to reduce 30% of the salt intake by 2025 (WHO, 2012), and countries have started to take it into account (Trieu et al., 2015; Webster et al., 2014; Webster et al., 2011; WHO, 2013; World Action on Salt & Health).

**TABLE 2:** Food challenges from salt reduction (Briggs et al., 2017).

Type of foods	Challenges and roles
Meat products	Reduces shelf life. Any further reduction may result in greater reliance on the preservative effect of salt
Bread and rolls	Low usage of rheological properties weakens the gluten and makes the dough sticky adversely influencing the efficiency of processing lines and causing wastage
Cheese	Salt influences various aspects of cheese quality such as texture, water binding capacity, and apparent viscosity, prevents undesirable microbial growth, influences aroma release, and supplies flavor
Cakes, pastries, and fruit pies	A higher level of salt is required in high‐viscosity products to maximize the taste of the flavor when compared with thinner products
Canned fish (salmon and tuna)	Canned tuna is frozen in brine for preservative purposes before putting it in oil, the water of brine. Hence, salt is added to thermally processed fish as a flavor enhancer as it influences texture and cooks yield

**TABLE 3:** Strategic approaches and current position for salt reduction (Briggs et al., 2017).

Strategies	Current position
Use of mineral salts	Currently, KCl is the feasible salt replacer because of its equivalent antimicrobial effect on pathogenic species. But its limitations lie in the pronounced bitter, chemical, and metallic taste and after taste, which is difficult to mask. Another example of mineral salts includes: milk salt (present in cheese); MgSO_4_ provides a salty and a bitter taste depending on the concentration and is a promising option for the future
Use of phosphate	These reduce NaCl concentration needed for the functionality of protein and control of water activity in meat products
Increased use of species	Another way salt can be reduced is the application of highly flavored species which has been successful in sauces to date. Other application includes soups and ready meals but not in bread and cheese
Use of taste enhancers	These are present in savory products which may influence consumers’ acceptability. Taste enhancers lack a salty taste but enhance salty taste when combined with NaCl. This is done by activating the receptors in the mouth and throat. Examples are amino acid, lactate, yeast products, etc

In another study (McMaster University, 2018), it was reported that sodium consumption does not increase health risks in the majority of individuals except for those who consume more than 5 g/day (2.5 teaspoons of salt). The result of their study revealed that the risk of heart disease and stroke is possible when a person's average daily salt is greater than 5 g. However, the health risk of salt consumption can be reduced or eliminated with the aid of a quality diet such as fruit, vegetables, potatoes, and other potassium‐rich foods. In a similar study, Oyebode et al. (2016) also mentioned that high sodium intake increases the risk of hypertension and cardiovascular diseases. Their study recommends that a maximum intake of 2 g/day as per WHO guidelines will result in a 30% reduction in sodium intake by 2025 in the population. The publication released by the WHO [24] listed key broad strategies for salt reduction. These include government policies (regulation to ensure that food manufacturers and retailers produce healthier foods), monitoring of population salt intake, creating an enabling environment for salt reduction through local policy intervention, and working with the private sector to improve the availability and accessibility of low salt intake. In a recent review, 59 of 83 countries mentioned have ongoing work programs with the food industry on reducing sodium in processed foods (Webster et al., 2021).

### 3.2 Fats

As part of the reason for food reformulation, reducing the intake of salts, sugar, and fat is always at the forefront. Reformulation of saturated fatty acid (SFA) and trans fatty acid (TFA) can also bring benefits to human health. Furthermore, in some studies (Silva et al., 2021) and Action Salt, UK (2020), it was revealed that banning all industrial TFA from processed food would avert 1700 to 7200 deaths. It is believed that fat reformulation would reduce deaths from cardiovascular diseases and cancer (Leroy et al., 2016). To reduce fat content and improve the overall fatty acid profile, there have been several approaches employed. Such fat reduction approaches deal with the changes in animal husbandry to produce learner animals and meat. They include manipulation of the diets of the dairy cow for the production of milk with less SFA, removal of fat during processing (trimming meat during slaughter), and skimming of milk to remove cream (Hadrová et al., 2021). Other approaches (see Table 1) include using baking technologies to change the profile of fatty acid pastry to reduce the content of fat in snacks, use of structured lipids, and blending of oil to avoid processes associated with the production of TFA during the production of margarine (Buttriss, 2013). Recent work addressed using traditional husbandry systems to produce better milk by supplying the animals with good‐quality green fodder that provides them with healthier nutrition and reduced disease and which can affect dairy production (Georges et al., 2019; Shamsuddin & Garcia‐Podesta, 2022). Other investigators examined the mechanisms used by dairy cows to provide milk through protein yield and improved quality of milk (Lapierre et al., 2021). In addition, a report Bates et al. (1999) revealed that SFA intake has fallen over the past couple of decades from 42 g in men and 31.1 g in women in the 1980s to 28.8 and 22.0 g/day, respectively, to date. Reformulation to reduce fat is a bit time‐consuming and cost‐effective due to the manufacturers retaining the characteristics of the products that are attractive to the consumers, which is part of the structural and organoleptic characteristics provided by SFA (see Table 4). Souza et al. (2015) reported the findings of a study conducted on both saturated and TFA consumption. It was observed that consumption of a higher amount of saturated fat was not associated with an increased risk when compared with a lower amount of health outcomes. In the report, it was noted that even though the consumption of a higher amount of TFA is associated with increased risk, the current guideline released by the WHO showed that saturated fatty acids (animal‐based foods such as meat and egg, as well as a plant‐based coconut, etc.) should be limited to less than 10% energy in the diet. This is not the case in Europe, where the consumption level is more than that. The TFA produced through the hydrogenation process contributes 1%–2% of the average diet. A cohort study conducted by Zhu et al. (2019) on the association between dietary fat intake and the risk of cardiovascular diseases (CVD) revealed that TFA intake was associated with increases in CVD. No association was observed between total fat, monosaturated fatty acids, and saturated fatty acid and the risk of CVDs. Considering the health concern of consumption of TFA, Bösch et al. (2021) stated that this can be consumed through baked goods, prepackaged food, and cooking oils. The major health risk of TFA as earlier seen has to deal with CVD, which has reported approximately 260,000 deaths, 6162, and 986 disability‐adjusted life years annually. Also, it increases the risk of death from any cause by 34% and coronary heart disease (CHD) by 28%. To reduce the rate of fat intake, the WHO recommended that total trans‐fat intake does not exceed 1% of total energy intake, which is about >2.2 g/day for a 2000‐calorie diet (World Health Organization, 2018). Interestingly, TFA can be replaced in food with the use of healthier fats and oils containing polyunsaturated or monounsaturated fats without affecting their consistency and taste (World Health Organization, 2019). The challenges faced in reformulating SFA reduction in selected food products for safety are presented in Table 4.

**TABLE 4:** Challenges affecting reformulation of reduction in SFA content (Cofrades & Alvarez, 2023).

Type of food	Challenges faced
Chilled bread fish range	Provision of the favorable ratio of unsaturated and saturated fatty acids. A better texture and eating quality were reported when a 16% reduction in SFA was achieved using rapeseed oil and also improved nutritional profile. However, this necessitated the development of a new breadcrumb which was a time‐consuming process
Frying oils	Change in blends affects change in the properties of the oil. Cheaper blends (higher in palm oil) are higher in SFA. In terms of optimal characteristics, there are good nutritional profile and long frying time without degradation. Minimal risk of polymerization (increases oil viscosity, oil absorption into fried product, and produces gums that stick to the fryer)
Margarine and spreads	SFA delivers the melting sensation in the mouth which is associated with butter. It provides a network of fat crystals that gives firmness to margarine and capture the liquid oil. Hence, reducing SFA results in a softer product during transport and storage. SFA also influences flavor release

### 3.3 Sugars

Sugar is known to be one of the targets for reduction in food reformulation, especially nonmilk extrinsic sugars (Bates et al., 1999). However, the World Health Organization (WHO) recommends that free sugar (monosaccharide and disaccharides) intake should be reduced to less than 10% of total daily energy in adults and children. A report released by the Institute of Medicine indicates that added sugar contains less than 25% of total calories (U.S. Department of Agriculture; U.S. Department of Health and Human Services, 2020). In the field of medicine, studies from epidemiologic and clinical traits revealed that individuals who consume a greater amount of added sugar (sugar‐sweetened beverages) tend to gain weight and face a risk of obesity, type 2 diabetes mellitus, dyslipidemia, hypertension, and cardiovascular diseases (Faruque et al., 2019; Schetz et al., 2019; Yang et al., 2014). A consensus about sugar is that it is a major contributing factor to the current obesity epidemic and diabetes, which may be due to the increase in products with high sugar content as well as the consumption of added sugar. This problem has been observed around the world in the last few decades. However, this has posed a challenge due to the function of sugar in foods that provides sweetness but suppresses sensations like bitterness and sourness. An experimental study recently conducted by Prada et al. (2022) reported that excessive sugar represents an increased risk of developing noncommunicable diseases (NCD). In the study carried out in Portugal, 1010 volunteers freely reported that all health conditions are associated with excessive sugar consumption, with the most risk being CMD, CVD, and mental health problems. In a similar study, Janzi et al. (2020) studied the association between added sugar intake and the risk of four different CVD in Sweden. Their finding revealed that added sugar intake of above 20% energy was associated with an increased risk of a coronary event when compared to the lower intake category.

The association between different added sugar sources and CVD has also been established in Africa. South Africa is regarded as the major consumer of sugar and the third most obese country in Africa. According to Myers et al. (2015), 40% of all death result from NCD, and sugar intake risk is one of them. They recommended the use of fiscal, regulatory, and legislative levers as methods or tools to reduce sugar consumption. Briggs et al. (2017) estimated that a reduction in sugar content of sugar‐sweetened beverages by 5 and 23% would reduce calorie intake by 4 kcal and 21 kcal/day, and Yeung et al. (2017) estimated a reduction in energy intake from 11 to 27 kcal/day if sugar content in selected sugar dense foods is reduced between 10% and 25%. One of the safety risks that may arise from reformulation is the presence of acrylamide in some types of bread (Gómez & Martinez, 2022) which is made at high temp above 120°C and low water content with reduced sugar. It is also found in coffee and fried potato products. It is 20 times less in French bread with concentrations of 19–30 ng/g (Becalski et al., 2003). Based on preclinical and clinical studies, high concentrations of sugars can lead to cognitive and memory impairment in rodents according to preclinical research (Spagnuolo et al., 2021).

Several studies (Hendriksen et al., 2011; Ma et al., 2016) highlighted a reduction in type 2 diabetes upon sugar reduction. The functions of sugar with their respective potential solutions are shown in Table 5. The approaches for reduction have limitations such as government regulatory constraints with high‐intensity sweeteners, gastrointestinal consequences associated with high intake of polyols, and resistance of many consumers to food containing lots of additives. In addition, alternatives are said to be more costly than sugar, which cannot replicate the unique flavor profile of sugar.

**TABLE 5:** Functions of sugar and potential solutions for sugar reduction.

Functions of sugar	Approaches for reduction
Sweetness	High‐intensity sweeteners
Color	Additive
Flavor	Additive
Stability /preservation	Additive
Bulk	Hydrocolloids, polyols, and dietary fiber

## 4 PERCEPTION OF CONSUMER RISK ON FOOD REFORMULATION AND CLEAR LABELING

Studies dealing with food product reformulation in the food sector have revealed a low number of inputs. On the other hand, studies have been conducted on the analysis of consumers' acceptance of reformulated food products. In Ratnayake et al. (2009), it was affirmed that the process and quality are the main challenges hindering reformulation based on the choices of the consumer. Young and Swinburn (2002) investigated the impact of a logo on food safety as an incentive for reformulation, which results in the consumer choosing a reformulated food product. The study focused on the consumer's choice and they concluded that the tick on approved products not only acts as a nutrition signpost for consumers but also can significantly influence the formulation of products without sacrificing taste or quality. Investigation of the analysis of reformulation practices in different countries suggests an examination role by the government (Traill et al., 2012). However, from van Raaij et al.' (2009) point of view, the practice of food reformulation is said to be limited by consumer acceptance, food safety, and the nature of the food products. However, consumers who read labels use less saturated fat and fewer calories (Kim et al., 2000). However, in reality, not all adults read product labels before buying food despite its advantage mentioned earlier. Meijer et al. (2021) explored whether the increasing number of label elements makes food useful, and impactful in conveying information to consumers, to help them make easier, safer, and healthier food choices and called for an increase in global harmonization in the use of label elements on foods. A comprehensive review (Bonsmann et al., 2020) of front‐of‐pack (FoP) labels have been carried out and it was suggested that around the globe, it appears that FoP schemes may affect reformulation efforts by the producer. An important way forward for product reformulation is the use of (FoP) labels, which may show healthier compositions and trends that are more favorable in nutrient content (Daphne et al., 2020) and enable regulators with information to establish an appropriate model (Santos et al., 2021).

### 4.1 Different approaches to food reformulation

**FIGURE 1:**
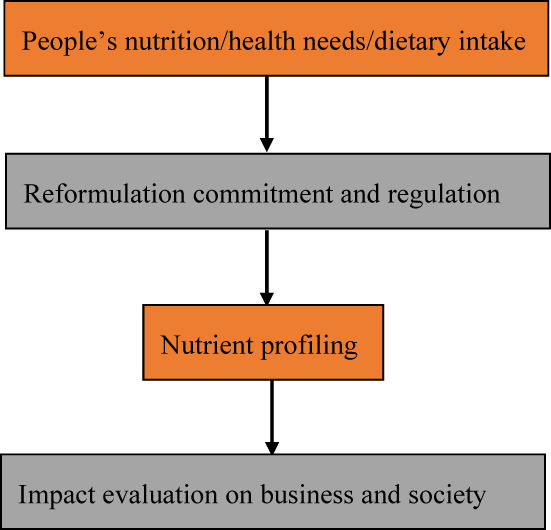
Approaches to food reformulation.

A four‐step methodological approach (Figure 1) to food production that promotes public health has been proposed (Spieldenner & van der Horst, 2018a, 2018b). The first step involves an analysis of people's nutrition, health needs, and dietary intake (protein, carbohydrates, vitamins, minerals, and water) followed by obtaining commitments and regulations for reformulation. The second step is about commitment to reformulation and regulations, whereas the third step deals with the improvement of food through food science, technology, and nutrient profiling, and the fourth step is the evaluation of the impact on business and society. The reformulation system can also be regarded as an integrated approach of four disciplines, namely food technology, legislation, nutrition, and health, and consumer perspectives (Van de Velde et al., 2016). It will take science, education, advocacy, and consumer demand to reformulate the food and beverage industry.

Using fortification and functionalization of existing food products are similar to reformulation, but they differ slightly. Fortification means adding micronutrients to foods or drinks (Olson et al., 2021), while functionalization refers to adding other good ingredients, specifically intended to improve the health of a food or beverage (Scrinis, 2016). Reformulation involves removing negative nutrients and ingredients and the addition of positive ingredients or nutrients to foods, ranging from minimally to highly processed (Table 6). Strategies suggested for the modification, functionality, and quality of food include using electrotechnologies for food‐structured systems (Pereira et al., 2023) and applying encapsulation and colloidal systems for delivering functionality (Dima et al., 2023). Aljuraiban et al. (2021) recommended that as we continue to chase the World Health Organization's target of 30% reduction in sodium intake, the quality of research, reducing bias in publications, and critical review of evidence need to improve.

**TABLE 6:** Types (Al‐Mashhadani et al., 2020) and approaches […] for food reformulation at different processing stages.

	Unprocessed foods	Minimally or moderately processed foods	Highly processed foods
Example foods	Vegetables, eggs, fruits	Fruits and dried vegetables, dairy products, grains, seafood	Bread, dairy products, breakfast cereals Chips, sugar‐sweetened beverages, cookies, crackers, cake, confectionery
Remove negative nutrients and ingredients	Not needed	Remove or lower salt from frozen or canned vegetables and fish, and remove or lower added sugar from dried or canned	Remove or lower added sugar, salt, and unhealthy fats Lower salt added sugar and unhealthy fats from all foods in this category but these foods should still be limited in the diet
Add positive nutrients and ingredients	Biofortification	Biofortification and fortification with minerals and vitamins, reformulation with added fiber, healthy fats, or protein	Fortification with minerals and vitamins, reformulation with added protein, fiber, or healthy fats

Reducing public health‐sensitive ingredients like sugar and salt has a limited application because reduction alone reduces volume and weight, which the consumer perceives as “less for more.” However, alternatives to sugar, such as other “fillers,” could be used to maintain volume and weight stability (Spieldenner & van der Horst, 2018a, 2018b). On the other hand, fillers like maltodextrin have physiological effects similar to sugar and are not recommended, even if they are allowed by most regulations. This is due to the effect it may have on diabetics regardless of the lower sugar effect. Another option is to change the physical structure of the food component itself. It is possible to reduce the “threshold” for sugar up to 37% in grape nectar and chocolates up to 29%. Sugar may also be replaced by sugar alcoholic products (e.g., maltitol and erythritol) and high‐intensity sweeteners (e.g., saccharin, stevia, sucralose, and aspartame acesulfame potassium) and fructan (i.e., inulin) or naturally produced sweeteners with a lower glycemic index (GI) but a greater capacity for sweetening (Gatta, 2015; Moriconi et al., 2020; Msomi et al., 2021). Reduced sugar consumption in drinks and yogurt is another intervention. Sugar improves the texture and volume of food. Added sugar can sometimes be simply removed. If reducing the amount of sugar is not possible, some food and beverage companies have introduced new light products that use artificial sweeteners (isomalt) instead of sugar. Many sugar‐free and sugar‐reduced foods are now available. Still, most of the time, the substitution results in some sort of compensation with other carbohydrates to keep the same volume of food (Harastani et al., 2020). Other recent breakthroughs in sugar reduction include the use of sourdough technology in cakes that permits the natural in situ production of polyols and the use of the newly structured, porous chocolate sugar (Di Monaco et al., 2018). For the chocolate sugar, it was highlighted that a lighter color and higher hardness are obtained if, for a healthy alternative, preparation is carried out with sap‐based sugar.

Numerous successful fat loss methods have been discovered. The Pickering technique, which is defined as “a technique in which emulsions are created mostly from healthy oils in the form of very small droplets covered by solid shells,” can be used to reduce fat in margarine and fat spreads (Harastani et al., 2020). The use of starch and Xanthan gum in sandwich spreads (mayonnaise) reduced fat by 30% (Gatta, 2015). A recent review looked at how carbohydrate, gum, and protein‐based coatings can help reduce oil uptake in deep‐fried foods (Morrison, 2015). Fat was decreased by 50% in sausages using oleogel rich in oleic acid. Fat was reduced by 60% in sausages using pork skin and green banana flour, and reduced by 32.8% in sausages using a gelled emulsion of linseed oil with carrageenan. Pectin gel was used as a 50% fat replacement in cheese (Lee et al., 2013). Salt reduction has been achieved by reducing particle size and causing a different distribution in the food product or food matrix. As a result of this, both governmental and nongovernmental bodies are working together to develop ways and ideas of reducing salt intake by 30% to achieve the WHO guideline of <5 g/day (Ismaeel et al., 2015). Medical literature data demonstrate a decrease in blood pressure as a result of a reduction in salt intake (Mente et al., 2014). Another way to cut costs is to improve taste perception by optimizing the surface of the nutrient and placing it in a more taste‐sensitive location. Salt crystals, for example, can be placed on the bottom of the pizza crust so that the particles immediately contact the tongue, causing the salty taste to be perceived. This is motivated as a result of the noncommunicable diseases associated with high intake of salt as earlier mentioned. To this effect, reducing salt in foods via crystal morphology dissolution properties of salt in the mouth has been proposed. Reports by Quilaqueo et al. (2015) revealed that crystal morphology might be variable in sodium reduction and salt intensity. Another option is to improve the nutrient as a whole—for example, replacing sodium with a combination of sodium and potassium chloride rather than sodium chloride alone can tackle widespread deficiency in potassium (Liberty et al., 2019). Excessive consumption of processed foods results in moderate chronic total body potassium depletion. Potassium helps the proper function of cardiovascular systems, kidneys, and bones (Lanham‐New et al., 2012).

### 4.2 Reductionist versus food matrix approach

Traditional reformulation of food has been mainly through the reductionist approach by reducing food components associated with health and well‐being. However, the manipulation of the food matrix is being proposed as the solution that will ensure that nutrients are delivered in the right amount while keeping away the undesirable components (Fardet & Rock, 2015). The debate in the literature shows advantages and disadvantages pointed out with mixed views in between. According to the report by Capuano and Oliviero (2018), the food matrix deals with the constituent of food and its structural organization at the microscopic, meso‐ and macrolevels. In addition, the reformulation of food based on the effect of the food matrix requires detailed knowledge of the physicochemical activities that take place in food. They posited that the reality is that this approach is mainly applicable to simple systems hence modeling based on data‐mining techniques and machine learning may elucidate chemical reactions in food matrixes. Acquisition of data based on the matrix effect has been carried out on different types of food components. The matrix structure has been redesigned to slow down digestion in starch‐rich products (Pellegrini et al., 2020), and chocolate structural properties of cocoa butter crystallization have been studied to reduce sugar and saturated fat (Ewens et al., 2021). An investigation showed that fat content in the food matrix could affect the structure, rheological properties, and digestive properties of protein (Ding et al., 2022). Specifically, leading to metabolic diseases such as obesity, hyperlipidemia, and diabetes. For instance, in a study conducted by Ding et al. (2022), four proteins (casein, pork, chicken, and soy protein) were investigated to find the influence of fat content on protein properties. The results showed that fat influences the solubility, hydrophobicity, and secondary structure of the protein. To be specific, under high‐fat conditions, the solubility of protein reduces and the content of α‐helix and β‐sheet increases. From the study, protein digestibility is usually affected by the type of protein and fat content. Hence, casein showed the highest protein digestibility of about 95%, followed by soy protein at about 89%. On the other hand, the increasing content of fat improved the digestibility of pork protein from 80% to 86% and chicken protein from 69% to 87%. It is interesting to state that the increased fat content did not affect the casein and soy protein (protein food) used in the study.

Other components of the food matrix and how it affects the availability of nanoparticles have also been studied (Hayder et al., 2022). Despite the progress in food matrix research, Aguilera (2018) view is that the term matrix is ambiguously used by investigators and therefore suggested a classification for the major types of food matrices. Examples of such food matrices include liquids (orange juice), gel (grape jelly), emulsion (mayo), fibrous materials (fruits and vegetables), crystals (sugar), and porous structures (marshmallows). These food matrices are regarded as a physical domain that tends to interact with specific constituents of food (nutrients), thereby providing functionality and behavior that are different from those exhibited by the components in isolation or free state (Aguilera, 2018). A good understanding of the food matrix will enable engineering to suit reformulation needs, but it is a well‐known fact that rearranging food matrixes is complex and its manipulation will require technology that poor manufacturers and small producers may not have (Harastani et al., 2020). Others have chosen the middle ground and suggested that sustainable future food systems may be aided by using a transdisciplinary integrative approach (Dietrich et al., 2021). This view is in line with the proposal that suggests a rational design that uses an integrated knowledge‐based versatile multidisciplinary system (Aguilera, 2022) to support food matrix reformulation.

For sustainable food matrix engineering, the integration of several disciplines (Ferranti, 2023) and the use of technological advances (Pérez‐Santaescolastica et al., 2021) have been suggested. Also, salt, fat, and sugar reduction must have consumer acceptance (O'Sullivan, 2020). Innovative exploration of the food matrix which can aid reformulation has been probed by other investigators. Reformulation may be differentiable (Telen et al., 2015) or personalized using 3D food printing (Aguilera, 2022; Zhang, Noort, et al., 2022; Zhang, Sun, et al., 2022). Oil structuring technology to obtain solid fats from liquid oils with a healthy lipid profile has been suggested by Cofrades and Alvarez (2023), and reformulation of food structures and functionalities to satisfy customers have been highlighted by Gormley (2019). Reformulation of healthy functional foods can help satiety (Munekata et al., 2021).

Food manufacturing industries face challenges to reduce energy density of food, glycemic response (Gomes et al., 2023), and incentives and disincentives to produce and commercialize high‐sugar products (Grasso et al., 2014). The limited effectiveness of voluntary agreements for sugar reduction. Deliza et al. (2021) but there has always been a campaign for using just‐about‐right scales in food product development and reformulation (Rothman, 2007) or a gradual reduction (Di Monaco et al., 2018). Investigators posit that process technologies in combination with other barriers to meet physicochemical, microbiological, sensory stability. A consensus is that reduction in dietary sodium (Dos Santos et al., 2023) and fat is required globally. A positive correlation between ready‐to‐eat food salt and fat has been found (Albuquerque et al., 2018). To improve future food research, ignorance and uncertainty will need to be removed (Knorr & Augustin, 2021) to aid reformulation by lowering fat, salt, and sugar content (Anderson et al., 2022). The position of Sadler et al. (2021) is that the “whole food” concept and the role of the food matrix will need more clarification and risk assessment.

The reductionist approach does not ever have ambiguity, which may sometimes follow the manipulation of the food matrix system. It simply involves reducing mainly the trio of salt, sugar, and fat in food to avoid noncommunicable diseases. Several governments around the world have taken this approach to improve the health of their citizens. In Europe, there are mandatory and voluntary provisions to reformulate and reduce the content of salts, sugars, and saturated and trans fatty acids in foods and beverages (Breda et al., 2020a, 2020b). We posit that the reductionist approach is still relevant even though reformulation by manipulating or engineering the food matrix can bring about tremendous beneficial results.

### 4.3 Challenges to food reformulation

According to Goryakin et al. (2019), a reformulation policy implemented properly can achieve positive health and economic outcomes for the consumers, public health, and the food industry. For instance, it reduces the risk of diet‐related noncommunicable diseases (obesity, type 2 diabetes, and cancer). Economically, reviewing and adjusting food reformulation are carried out by manufacturers to adapt to consumer preferences. In so doing, lowering the production costs and increasing profit (Gressier et al., 2020). Despite the advantages and benefits derived from food reformulation, such as nutritional and health benefits (Botez et al., 2017), every food business operator faces reformulation challenges such as increased numbers of ingredients, increased warnings on packs, insignificant changes to the content of energy products, and ingredients that consumers are not familiar with, as well as the potential impact of food safety (Copper, 2017). This can be a change in resources when an ingredient becomes unavailable or unaffordable to the alteration of the recipe due to consumers' wishes and the need for innovation (Staudigel & Anders, 2020). The reduction in salt, fat, and sugar poses significant challenges due to the integral nature of the ingredient and the many functions they serve within a product. Völkl (2018) commented that changes to the food products composition affect technical processing properties, product quality and safety, shelf life, sensory properties, and consumer acceptance. The reaction of some governmental measures toward food reformulation possesses a challenge. For instance, the response of food manufacturers to the labeling requirement. This action may make the product more attractive to consumers, thereby reducing calorie, salt, or sugar content (Hawley et al., 2013). Also, taxation of sugary beverages and other calorie‐dense product induce product reformulation which often increases or have a positive impact on health. In addition, unjustified health claims are sometimes made or occur during the process of food reformulation (Chaloupka et al., 2019). Another aspect has to deal with the manufacturer whereby they frequently reformulate their products in response to changes in ingredient prices, the availability of common supplies, or, more recently, government or consumer demands for reduced sugar, salt, or fat. How all these trends overlap, such as health and well‐being promotion products with small amounts of sugar, fat, and salt, underlines the major challenges that most companies face in reformulation. Reformulating the product's taste, texture, appearance, functionality, safety, and shelf life while maintaining the remainder of competitive prices with entirely natural ingredients can be a major task (Komitopoulou & Gibbs, 2012). Although the above‐mentioned sugar, fat, and salt reduction opportunities are extensive, it is challenging to reformulate food because of specific restrictions. Reformulated products are still required to comply with applicable laws. It is still possible to reformulate any type of food if the end product conforms to the food requirements and directives. A bigger challenge is encountered where alterations to the recipe and method of production are not allowable for some traditional products that are protected by law (Harastani et al., 2020).

### 4.4 Risks and limits of reformulation

Before any food reformulation, a thorough risk analysis, risk assessment, and effective risk management must be carried out. It is critical to have a thorough understanding of a product and what each ingredient does and contributes to the formulation. Only then will it be possible to determine the safety limits and ensure that any proposed reformulation will result in a safe and stable product. New food packaging concepts from traditional methods have been reviewed (Salgado et al., 2021) recently and it was highlighted that intelligent packaging can preserve shelf‐life among other innovations. Also, if required, features like consumer acceptance, regulatory aspects, and scale‐up costs are diligently considered, food packaging can be reshaped in future (Chaudhary et al., 2022).

Reformulation can cause fatal food poisoning outbreaks, when you remove fats, sugar, and salt from food, the water activity in the food goes up. This impacts the ability of bacteria to grow in the food to a level that leads them to cause food poisoning, otherwise, the shelf life of the food has to be reduced to make sure bacteria cannot get to that level (Stones, 2014). Similarly, the combinations of intrinsic factors (pH, salt, sugar, and preservatives) actively contribute to food safety and shelf life in a wide range of foods (Komitopoulou & Gibbs, 2012). To avoid food spoilage, industries have had to find an effective replacement for salt capacity due to sodium reduction in one product. Another issue is obtaining healthier nutrients through appropriate technological support, which guarantees the structure of food with alternative ingredients that replace saturated fat, for instance. The question of whether or not what replaces the substituted product is necessarily better for health is relevant (Traill et al., 2012). When private companies replace trans fat with saturated fat, the combined content of these fats in the food may remain about the same or even increase, posing a risk to consumers (Mozaffarian & Jacobson, 2010). When it comes to soft drinks, where sugar has been reduced due to the use of sweetening agents, there are still some concerns about the long‐term health effects of diet.

## 5 AGENTS COMPLEMENTARY TO IMPLEMENTING SUCCESSFUL FOOD REFORMULATION INTERVENTION

### 5.1 Consumer acceptance, food companies, and government policies

Consumers have the potential to receive or change reformulated foods according to their personal best interests, tastes, or appreciation. For example, consumers can see the “new” product as too different from the previous one in terms of taste and may conclude that it does not please them and they buy something else. On the other hand, consumers may decide that natural (fat and sugar) ingredients are better than artificial ones. Rising prices can also influence consumers, as healthy foods may require costlier input and processing and they might not be prepared to spend more for healthier choices. Another option has to do with psychology. Indeed, if buyers decide that the product reformulated is healthier but has no flavor, they will move on to other food items because of the perception that “low fat has poor flavour” (Gatta, 2015). Interestingly, it is worth knowing that increasing consumers' awareness of the relationship between food and health and improving consumers' food environment promotes and facilitates healthier diets or choices. This is done through information and education campaigns (Brambila‐Macias et al., 2011; Mazzocchi et al., 2012). Changing food composition takes time and investment, industries prefer to market a brand‐new product rather than reformulating “old” food products. Costs of food reformulation to ensure consumers' taste, texture, and safety are high and vary according to the type of products, enterprises, and techniques used. Food companies can “reformulate products in a way that may justify health claims” due to government regulations, which can open up new market opportunities (Sassi, 2010).

Governments play a key role in boosting and promoting the reformulation of foodstuffs. Laws that define compositional standards for certain foods or selected parts of the population have already been established. In 2005, for example, the British administration launched a program to reduce salt in food industries, which influenced certain nutrient levels in certain products. A further option is to set criteria for a specific population, for example, reducing the consumption of fat in school foods. In addition, industries can be encouraged to change the composition of foods with healthier ingredients in cooperation with governments (Gatta, 2015). Many countries, especially public health department organizations, implement partnerships with the food industry and retail sector as a control measure to improve the nutritional quality of foods available in the market based on food reformulation (Chauliac & Hercberg, 2012; Combris et al., 2011; Hendry et al., 2015; Trevena et al., 2014).

Reformulating existing and commonly used processed foods can be a very realistic approach and opportunity for improving people's health and planetary health with a significant positive impact estimated on obesity (Spieldenner & van der Horst, 2018a, 2018b). Food reformulation is attractive because it calls for the least change in consumer diet. The reformulation of food over time with progressive changes may minimize consumer perceptions and negative attitudes when both taste and palatability are maintained, thus maintaining unchanged food buying and eating patterns. The vast majority of consumers would not notice the gradual reduction of “baddies” (sugar, sodium, saturated, and trans fats) ingredients (Gatta, 2015). The reduction of sodium can affect the food safety in the reformulated products, and hence, the knowledge of the food consumption habit should be characterized (Ana Gomes et al., 2023). While reformulation could ultimately reduce intakes of individual foods, it could enhance the nutritional quality of processed foods not only by decreasing “baddies” but also by contributing to positive nutrients, such as vitamins and minerals, in diets and enabling low‐cost food fortification to enhance nutrient density (Spieldenner & van der Horst, 2018a, 2018b).

Processed and prepackaged foods are an important part of diets worldwide, accounting for more than half of calorie intake, thus playing a critical role in human diets. Dietary intake and eating behavior research help clarify the types of foods that need to be reformulated; first by identifying the foods consumed by the majority of a population and in the greatest quantities (Spieldenner & van der Horst, 2018a, 2018b). An analysis (Staudigel & Anders, 2020) of the Food and Drug Administration of the United States on reduction strategy for sodium in chip products found that intake was reduced by up to 7%. Silva et al. (2021) reviewed a decade's evolution of taxation on sugary soft drinks and the use of artificial sweeteners in Portugal and found that sugar content has reduced even though reformulations did not succeed to reduce the intensity of sweet taste. In the United Kingdom, a report (Action Salt, UK, 2020) of a trend analysis showed that there has been no change in salt intake since 2009, indicating the need for drastic action. In that report, it was highlighted that salt reduction was not the only concern, hence, to reduce the intake of salt, sugar, and fat, the government imposed a ban on TV and online adverts for food high in fat, sugar, and salt before 9 pm. The ban included no promotions of unhealthy food high in salt, sugar, and fat, and the requirement for calories to be displayed on menus to help people make healthier choices when eating out. Copying the UK model, the National Salt and Sugar Reduction Initiative was started in the United States (National Salt and Sugar Reduction Initiative (NSSRI), 2018). Around the world, health authorities drive citizens to change their lifestyles by reducing the intake of salts, sugars, and fats.

### 5.2 Food reformulation by fortification in developing countries

The partnerships between the food industry and retail sector found in the developed world are not replicated exactly in developing countries. Reformulation of food around the developed world can have the desired effect because the majority of people eat processed food purchased from supermarkets. Therefore, any change in food content can be rolled out for millions of consumers. In developing countries, an estimated 58% of the population lives in rural areas (World Bank, 2020) and most of these people have very low economic power and do not rely on supermarkets for their food. Food eaten by families in rural areas is mainly artisanal food (e.g., processed cassava), which is mainly starchy (Nwaiwu & Onyeaka, 2022) or low in other essential nutrients. This problem has been recognized and the consensus is that at a stage during processing, low‐nutrient foods can be reformulated by fortification with relevant nutrients. Fortification of starchy cassava (*Manihot spp*.) granules during processing with peanuts high in protein caused a reduction in the carbohydrate content by 10% (Arisa et al., 2011). Also, an investigation (Alozie & Ekerette, 2017) found that when it was fortified with soybeans, melon seed, and moringa seed flours, the nutritive values and physicochemical and sensory properties improved. Another approach is the use of lactic fermentation as a strategy to improve the nutritional and functional values of pseudocereals (Rollán et al., 2019). As explained, this involves the capacity of these bacteria to decrease antinutritional factors such as phytic acid while ensuring that the functional value of phytochemicals such as phenolic compounds and B‐group vitamins are increased. Many developing countries, especially Africa, rely on indigenous fermented foods (Onyeaka et al., 2022), hence a closer study of the fermentation process and the microorganisms involved may help reformulate the food. There are food safety concerns, especially with microorganisms (Nwaiwu et al., 2020) and chemical contaminants (Nwaiwu & Itumoh, 2017), and these need to be taken into consideration. There is a possibility that other nutrients may be lost if the natural structure of food material is tampered with. Concern was expressed (Charlton et al., 2018) when South Africa deliberated on a mandatory salt reduction policy. It was suggested it could result in less intake of iodine and affect the iodization program which encouraged the consumption of iodized salt. A pediatric study (Iacone et al., 2021) of up to 1000 children found that iodine intake from iodized salt was up to 20%. The conclusion from that study was that health institutions should continue to support iodoprophylaxis. A way to increase the availability of key nutrients may be through the use of nanotechnology approaches and genetic biofortification as suggested by others (Ohanenye et al., 2021).

## 6 CONCLUSIONS

The consumption of excessive amounts of energy, salt, saturated fat, and sugar can lead to an increased risk of chronic diseases such as cardiovascular disease, cancer, and diabetes. Nutrition and health are, therefore, crucial drivers in the food reformulation process. Reformulation can improve diets, reduce the negative consequences of consuming certain foods, such as obesity and nutrition‐related diseases, and increase nutrient intake in the diet. This is achieved without the need for consumers to make major changes to their eating habits. However, it is important to note that food reformulation cannot replace a healthy diet, and some foods will always be considered unhealthy options. Effective dietary changes can only be achieved through a combination of voluntary measures, regulations, and individual behavioral changes. Voluntary reformulation by the food industry alone is not enough, and evidence suggests that it should be combined with food labeling and public awareness campaigns (food education) to increase awareness and expand options.

The food reformulation process is influenced by factors such as food technology, nutrition and health, legislation, and consumer perspectives. Successful implementation of food reformulation policies requires the collaboration of all parties, including the food industry, government, and consumers. Effective information campaigns can drive change in food composition and provide a competitive advantage for businesses. In conclusion, the food reformulation policy is likely to succeed if the private sector works in partnership with and responds to government pressure. Future research should focus on investigating the challenges, opportunities, and health and economic benefits of reformulating commonly consumed processed foods with nutrients other than sodium (saturated fat, trans fat, total energy, whole grains, fruits and vegetables, and fiber).

## CONFLICT OF INTEREST STATEMENT

The authors declare that the research was conducted in the absence of any commercial or financial relationships that could be construed as a potential conflict of interest.

## Data Availability

Data sharing is not applicable to this article as no new data were created or analyzed in this study.
